# Manual hyperinflation attenuates reduction of functional residual capacity in cardiac surgical patients: a randomized controlled trial

**DOI:** 10.1186/cc9428

**Published:** 2011-03-11

**Authors:** F Paulus, DP Veelo, SB De Nijs, P Bresser, BA De Mol, LF Beenen, JM Binnekade, MJ Schultz

**Affiliations:** 1 Academic Medical Center, Amsterdam, the Netherlands

## Introduction

Cardiac surgical patients show deterioration of functional residual capacity (FRC) after surgery. Manual hyperinflation (MH) aims at preventing airway plugging, and as such could prevent the reduction of FRC after surgery. The purpose of this study was to determine the effect of MH on FRC in cardiac surgical patients.

## Methods

This was a randomized controlled trial of patients after elective coronary artery bypass graft and/or valve surgery admitted to the ICU of a university hospital. Patients were randomly allocated to routine MH strategy (MH within 30 minutes after arrival in the ICU and every 6 hours until tracheal extubation) or on-demand MH (MH only in cases of perceptible (audible) sputum in the larger airways or in case of a drop in SpO_2_) during mechanical ventilation. The primary endpoint was the change of FRC from the day before cardiac surgery to 1, 3, and 5 days after tracheal extubation. Secondary endpoints were SpO_2_, on the same time points, and chest radiograph abnormalities at day 3.

## Results

**Figure 1:**
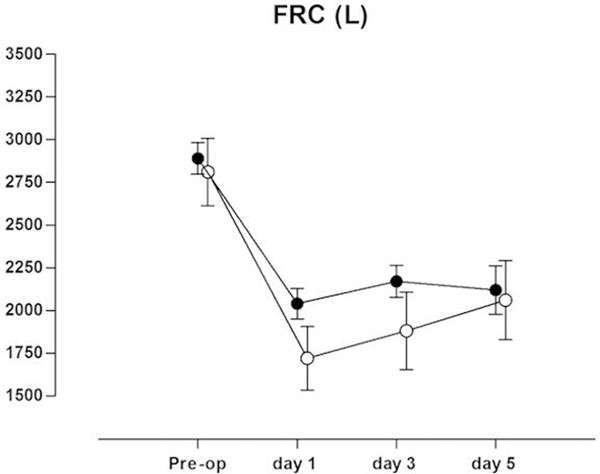
**Pulmonary function measurements**. Preoperative functional residual capacity (FRC (l); mean, 95% CI) and FRC at 1, 3, and 5 days after extubation in the routine MH group (closed circles) and in the on-demand MH group (open circles).

One hundred patients were enrolled. In the routine MH group FRC decreased to 72% of the preoperative measurement, versus 59% in the on-demand MH group (*P *= 0.002). Differences in FRC were not longer statistically significant at day 5 (Figure 1). There were no differences in SpO_2 _between the two groups. Chest radiographs showed more abnormalities in the on-demand MH group compared with patients in the routine MH group (*P *= 0.002).

## Conclusions

MH attenuates the reduction of FRC in the first three postoperative days after cardiac surgery.

